# Subclinical thyroid dysfunction and the risk of incident atrial fibrillation: A systematic review and meta-analysis

**DOI:** 10.1371/journal.pone.0296413

**Published:** 2024-01-02

**Authors:** Hasveer Singh, Mariam Z. Shahid, Stephanie L. Harrison, Deirdre A. Lane, Gregory Y. H. Lip, Sunil Jit R. J. Logantha

**Affiliations:** 1 Liverpool Centre for Cardiovascular Science at University of Liverpool, Liverpool John Moores University and Liverpool Heart & Chest Hospital, Liverpool, United Kingdom; 2 Department of Cardiovascular and Metabolic Medicine, Institute of Life Course and Medical Sciences, University of Liverpool, Liverpool, United Kingdom; 3 Geriatric Medicine, Airedale General Hospital, Airedale Hospital Trusts, Bradford, United Kingdom; 4 Danish Center for Health Services Research, Department of Clinical Medicine, Faculty of Health, Aalborg University, Aalborg, Denmark; Albert Einstein College of Medicine, UNITED STATES

## Abstract

**Background:**

Thyroid hormones act on the cardiovascular system directly by modulating its function and indirectly by transcriptional regulation of gene expression in the heart and the vasculature. Studies have shown associations between overt and subclinical thyroid disorders and cardiovascular outcomes. The aim of this study was to perform a systematic review and meta-analysis to assess the potential relationships between subclinical hyper- and hypothyroidism and risk of atrial fibrillation (AF), and post-operative AF.

**Methods:**

MEDLINE and Scopus databases were searched from inception to 18^th^ February 2023 for randomised controlled trials, case-control studies, and cohort studies which assessed the relationship between subclinical thyroid dysfunction and incident AF events. Risk of bias and the quality of evidence were assessed using the RoBANS tool and GRADE approach, respectively. Meta-analysis was conducted in Review Manager 5.4 using the Mantel-Haenszel statistical method and a random-effects model. Data are presented as risk ratios with 95% confidence intervals. Statistical heterogeneity amongst studies was assessed by the chi-squared (χ^2^) test and *I*^2^ statistic. *p*≤0.05 were considered significant.

**Results:**

A total of 6467 records were identified, of which 10 cohort studies met the inclusion criteria. Both subclinical hyperthyroidism and subclinical hypothyroidism were associated with an increased risk of incident AF (risk ratio (RR), 1.99; 95% confidence interval (CI), 1.43–2.77; n = 5 studies; *p*<0.0001 and RR, 1.19; CI, 1.03–1.39; n = 7 studies; *p* = 0.02, respectively). Subgroup analysis for post-operative AF revealed marked heterogeneity between studies (*I*^2^ = 84%) and association with subclinical hypothyroidism was not significant (RR, 1.41; CI, 0.89–2.22; n = 3 studies; *p* = 0.15).

**Conclusions:**

The current evidence suggests that both subclinical hyperthyroidism and subclinical hypothyroidism are associated with increased risk of incident AF. Further investigation is required to determine potential causal links that would guide future clinical practice.

## Introduction

Thyroid dysfunction is a common endocrine disorder known to exacerbate several cardiovascular pathologies including arrhythmia, dyslipidaemia, and heart failure [1]. Overt hyperthyroidism is a well-established risk factor for the development of atrial fibrillation (AF) and overt hypothyroidism is associated with ventricular arrhythmias [2, 3]. However, there is a lack of consensus on the associations between mild, early forms of thyroid dysfunction (subclinical) and AF.

Subclinical thyroid dysfunction is a primary disease of the thyroid glad defined by abnormal serum thyroid stimulating hormone (TSH) and normal free tetraiodothyronine (T4) and free triiodothyronine (T3) levels [4]. Subclinical hyperthyroidism (low TSH and normal T4) and subclinical hypothyroidism (high TSH and normal T4) affect 4–10% and 2% of the general population, respectively [5]. Women and older adults are primarily affected [6]. About 5% of the European population with subclinical thyroid dysfunction remains undiagnosed [7, 8]. The National Institiute for Health and Care Excellence (NICE) guidelines in the UK recommend treatment for subclinical hypothyroidism with levothyroxine if TSH levels are consistently >10 mIU/L or if consistently above normal reference range with symptoms [9]. Currently, no standard treatment is recommended for subclinical hyperthyroidism [9]. Additionally, suboptimal treatment for thyroid dysfunction is common and increases risk of incident AF, stroke, and cardiovascular mortality [10, 11]. In this context, a systematic review and meta-analysis to identify potential associations between subclinical thyroid dysfunction and AF would be valuable for framing guidelines and evolving clinical practice.

AF is the most common arrhythmia and in 2017 there were 37.57 million prevalent cases and 3.05 million incident cases of AF globally [12]. AF is associated with an increased risk of mortality [13], dementia [14], and stroke and heart failure [15, 16]. Post-operative AF affects 40% of cardiac surgery patients, more than doubles stroke risk, and increases the length of hospital stay [17, 18]. Mortality during admission and 6 months post-operatively is significantly increased in patients with post-operative AF [19]. Hence, there is a significant health and financial burden attached to developing AF per se, and post-operatively.

Several studies have demonstrated the effects of subclinical thyroid dysfunction on adverse cardiovascular outcomes, including coronary heart disease [20], atherosclerosis and myocardial infarction [21], and heart failure [22]. Given the associations between thyroid dysfunction and cardiovascular outcomes and the potentially modifiable nature of thyroid dysfunction, we performed a systematic review to determine whether subclinical thyroid dysfunction is associated with a higher risk of incident AF per se and in post-operative patients.

## Methods

This systematic review was prospectively registered on PROSPERO (CRD42020221565) and reported in accordance with Preferred Reporting Items for Systematic Reviews and Meta-Analyses (PRISMA) guidelines.

### Information sources and search strategy

MEDLINE and Scopus databases were searched from inception to 18^th^ February 2023 without language restrictions. The search terms used in the databases as keywords were: “subclinical hyperthyroidism”, “subclinical hypothyroidism”, “subclinical thyroid”, “thyroid dysfunction”, “thyroid”, “atrial fibrillation”, and “arrhythmia”. These were combined using the Boolean operators “AND” and “OR”. The full search strategy for both databases is provided in S1 Table. Reference lists of included studies were searched for additional relevant literature. Results were imported into EndNote X9 (Clarivate Analytics), and duplicate records removed.

### Eligibility criteria

Studies which measured AF incidence in patients with subclinical thyroid dysfunction were included. Systematic reviews were screened for additional studies relevant to the study question. The following inclusion criteria applied: 1) cohort studies, case-control studies, RCTs, systematic reviews and 2) thyroid function tests, including serum TSH and T4, taken at baseline to determine subclinical thyroid dysfunction. The exclusion criteria were: 1) studies involving non-human subjects 2) no measurement of serum TSH and T4 at baseline prior to AF diagnosis 3) AF diagnosed prior to index hospitalisation 4) not including AF as an outcome and 5) review articles or cross-sectional studies.

Studies were included if it could be determined which participants did not have prevalent AF at baseline. Cross-sectional studies and observational studies where thyroid hormone levels were measured after AF was diagnosed were excluded as incidence of new-onset AF could not be ascertained.

### Study selection

Two reviewers (HS, MZS/SJRJL) independently screened a 25% sample of the records, 1266 records in total, based on their title and abstract. Agreement at this stage was 99.6% and disagreements were resolved through discussion. As per the PROSPERO protocol, with agreement >80%, the primary reviewer (HS) screened the remaining records to determine inclusion for full text screening. Studies from previous systematic reviews were extracted during the selection process. The same two reviewers (HS, MZS/SJRJL) independently screened the full texts to determine final inclusion for synthesis. Agreement at full-text screening was 94.1% and disagreements were resolved through discussion. Studies in which a euthyroid control group was included and hence a risk ratio (RR) could be calculated were selected for meta-analysis.

### Data extraction

The primary reviewer (HS) independently extracted data from the selected studies into a predesigned data extraction form on Microsoft Excel. The second reviewer (MZS) verified the extracted data; there were no disagreements. Data extracted included authors, year of publication, journal name, study design, number of participants, follow-up period, participant age, participant gender, TSH values used, method of AF ascertainment, previous history of cardiovascular disease, incidence of AF and the other outcomes measured. We contacted authors of six studies for further information; however, no data was provided.

### Risk of bias of included studies

The risk of bias was assessed by two reviewers (HS, SJRJL) independently using the Risk of Bias Assessment tool for Non-Randomised Studies (RoBANS) [23]. Each study was assessed under the six RoBANS domains (selection of participants, confounding variables, exposure measurement, blinding of outcome assessment, incomplete outcome data and selective outcome reporting) and then the risk of bias was classified as low, high, or unclear as per pre-set criteria (S2 Table). The overall risk of bias for the studies was determined as low if ≤1 domain was classified as high or unclear risk, medium if 2 domains were classified as high or unclear risk, and high if ≥3 domains were classified as high or unclear risk. Non-consideration of the effect of baseline thyroid medication was considered a confounding factor. The risk of bias was assessed at the outcome level of AF and not for the entire study.

### Assessing the quality of the evidence

Quality was independently assessed by two reviewers (HS, SJRJL) using the Grading of Recommendations, Assessment, Development and Evaluations (GRADE) [24]. This assessed the risk of bias, imprecision, inconsistency, indirectness, and publication bias of the studies. Outcomes were graded as high, moderate, low, or very low certainty. Summary of findings tables were generated for AF, non-post-operative AF, and post-operative AF, where applicable, using GRADEpro GDT [25].

### Data synthesis and statistical analysis

Results of studies without a control group were expressed as the proportion with subclinical thyroid dysfunction who developed AF. Meta-analysis was conducted in RevMan, Version 5.4 [26]. The Mantel-Haenszel statistical method, random-effects analysis model was used, and the effect size is presented as RR with 95% confidence intervals (CI). Forest plots were used to depict the effect of subclinical thyroid dysfunction on incident AF. Statistical heterogeneity amongst studies was assessed by the chi-squared (χ^2^) test and *I*^2^ statistic using RevMan [26, 27] and *p*-values≤0.05 were considered significant.

### Differences between protocol and review

In the prospectively registered PROSPERO protocol (CRD42020221565) we defined subclinical thyroid dysfunction as a TSH value outside the normal range of 0.45–4.50 mU/l and T3 and/or T4 values within their normal range. During the review we identified varying definitions for subclinical hyperthyroidism and subclinical hypothyroidism between studies. Excluding studies that did not strictly adhere to the TSH range specified in the PROSPERO protocol would have significantly reduced the total number of included studies and meta-analyses would not be possible. Therefore, this review included studies with varying definitions of subclinical thyroid dysfunction, as defined by the respective study.

Pre-planned subgroup analyses examining gender and subclinical thyroid dysfunction and timing of the onset of incident AF could not be undertaken due to lack of sufficient data. A subgroup analysis for incidence of post-operative AF and non-post-operative AF was performed. Sensitivity analysis was performed to identify sources of heterogeneity.

## Results

### Study selection

**Fig 1:**
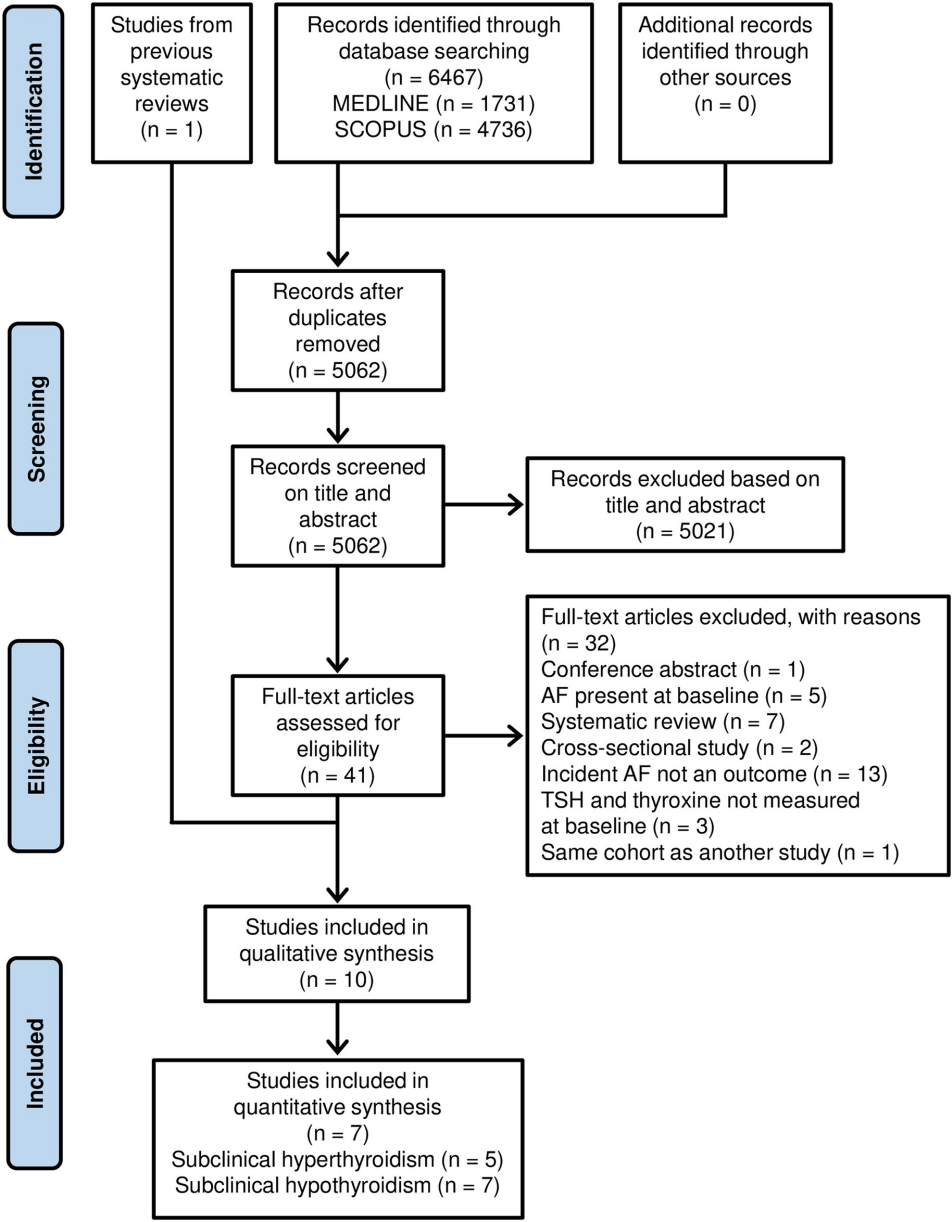
PRISMA flow diagram. Study selection process for this systematic review.

MEDLINE and Scopus searches returned 6467 records and after removing 1405 duplicates, 5062 records were screened based on title and abstract, with 5021 (99%) records excluded for either not fulfilling inclusion criteria or not being relevant to the review question. Of the 41 remaining records, seven were systematic reviews, of which one additional study was identified. Thus, full text articles of 42 studies were assessed and 10 studies were included (Fig 1) [28–37].

### Study characteristics

Study characteristics are summarised in Table 1. Of the 602,257 participants in the identified studies, 596,358 participants were included in this systematic review; 6,732 participants had subclinical hyperthyroidism, 13,628 had subclinical hypothyroidism and 575,998 were euthyroid. There were 19,263 cases of incident AF. Follow-up length ranged from post-operative [30, 35, 37] to 13.2 years [36]. Mean age ranged from 48.9 to 75.3 years [33, 34]; mean participant age was >65 years in five studies [29–31, 33, 35]. All included studies were cohort studies. Three studies involved patients undergoing cardiac surgery [30, 35, 37]; patients in Park *et al*. [30] and Zhao *et al*. [37] underwent coronary artery bypass graft (CABG) surgery and those in Martínez-Comendador *et al*. [35] had undergone aortic valve replacement procedure. For subclinical hyperthyroidism, TSH values used in these studies were <0.10 [28] to <0.45 [33] mIU/L and for subclinical hypothyroidism >3.40 [36] to >5 [34] mIU/L. The British Thyroid Foundation normal range for TSH is 0.4–4.0 mIU/L and those for free T4 and free T3 are 9.0–25.0 pmol/L and 3.5–7.8 pmol/L, respectively [38].

**Table 1:** Characteristics of included studies.

Study	Study design	Number of participants	Age (years)	Women (%) †	TSH cut-off value (mIU/L)	Follow-up	Thyroid medication at baseline	Incident atrial fibrillation
Tenerz *et al*. 1990 [28]	Prospective Cohort	Total: 80; SH: 32; SCH: NA; Euthyroid: 36	Mean 65	87.5	SH: < 0.10; SCH: NA	2 years	Not reported	SH: 3; SCH: NA Euthyroid: 0
Cappola *et al*. 2006 [29]	Prospective Cohort	Total: 3233; SH: 43; SCH: 472; Euthyroid: 2502	Mean 72.7	59.6	SH: < 0.44; SCH: 4.50–20.00	Mean 12.5 years	No	SH: 22; SCH: 142 Euthyroid: 703
Park *et al*. 2009 [30]	Prospective Cohort	Total: 260; SH: NA; SCH: 33; Euthyroid: 214	Mean 65.3	30.0	SH: NA SCH: > 4.1	Post-operative	Not reported	SH: NA; SCH: 15 Euthyroid: 64
Rosario 2010 [31]	Prospective Cohort	Total: 102; SH: 102; SCH: NA; Euthyroid: NA	Median 68	100.0	SH: 0.10–0.40; SCH: NA	Median 3.4 years	No	SH: 3; SCH: NA Euthyroid: NA
Poola *et al*. 2011 [32]	Retrospective Cohort	Total: 116; SH: 108; SCH: NA; Euthyroid: NA	Mean 55.0	75.9	SH: < 0.40; SCH: NA	Median 3.2 years	No	SH: 3; SCH: NA Euthyroid: NA
Nanchen *et al*. 2012 [33]	Prospective Cohort	Total: 5316; SH: 71; SCH: 199; Euthyroid: 5046	Mean 75.3	50.5	SH: < 0.45; SCH: ≥ 4.50	3.2 years	Yes	SH: 3; SCH: 16 Euthyroid: 478
Selmer *et al*. 2012 [34]	Retrospective Cohort	Total: 586460; SH: 6276; SCH: 12087; Euthyroid: 562461	Mean 48.9	60.6	SH: < 0.20; SCH: > 5.00	Median 5.5 years	No	SH: 435; SCH: 402 Euthyroid: 16275
Martínez-Comendador *et al*. 2016 [35]	Prospective Cohort	Total: 467; SH: NA; SCH: 35; Euthyroid: 432	Mean 70.6	40.7	SH: NA; SCH: > 4.10	Post-operative	Not reported	SH: NA; SCH: 20 Euthyroid: 129
Langén *et al*. 2018 [36]	Prospective Cohort	Total: 5133; SH: 100; SCH: 257; Euthyroid: 4762	Mean 50.9	53.0	SH: < 0.40; SCH: > 3.40	Median 13.2 years	No	SH: 15; SCH: 21 Euthyroid: 291
Zhao *et al*. 2021 [37]	Retrospective Cohort	Total: 1090; SH: NA; SCH: 545; Euthyroid: 545	Median 64	37	SH: NA; SCH: > 4.78	Post-operative	No	SH: NA; SCH: 111 Euthyroid: 112

NA: not applicable; SH: subclinical hyperthyroidism; SCH: subclinical hypothyroidism.

^†^ Represents the proportion of women in each study.

### Subclinical hyperthyroidism and incidence of AF

**Fig 2:**
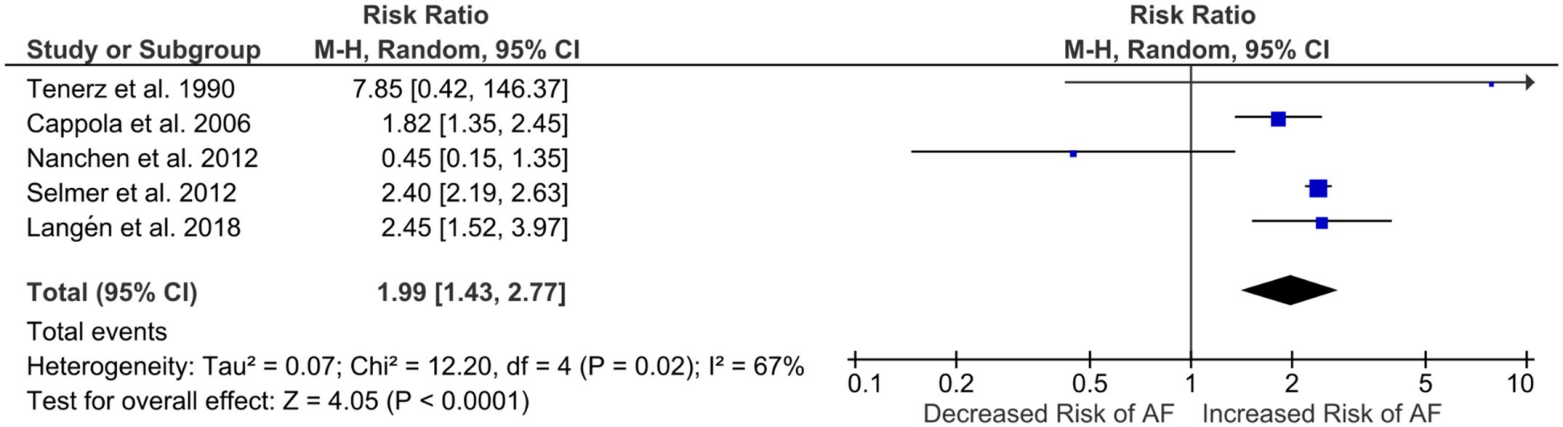
Forest plot of risk ratio for incident atrial fibrillation in patients with subclinical hyperthyroidism. Risk ratios for individual studies and the overall effect sizes are shown. The size of blue squares corresponds with the weighting of the study and bars represent 95% confidence intervals. Risk ratio expresses the risk of atrial fibrillation (AF) occurring in patients with subclinical hyperthyroidism relative to euthyroid.

The effect of subclinical hyperthyroidism on the incidence of AF was assessed in seven studies [28, 29, 31–34, 36]. Among 6,732 patients with subclinical hyperthyroidism in these studies, 484 cases of incident AF were reported. Two studies [31, 32], did not have a control euthyroid group, 2.94% and 2.78% of patients in these two studies developed AF at follow-up, respectively [31, 32]. The remaining five studies were eligible for meta-analysis and the risk of incident AF in patients with subclinical hyperthyroidism relative to euthyroid (RR) was 1.99 (95% CI, 1.43–2.77; *p*<0.0001) (Fig 2) [28, 29, 33, 34, 36]. There was substantial heterogeneity between studies (*I*^2^ = 67%; see section on sensitivity analysis).

## Subclinical hypothyroidism and incidence of AF

**Fig 3:**
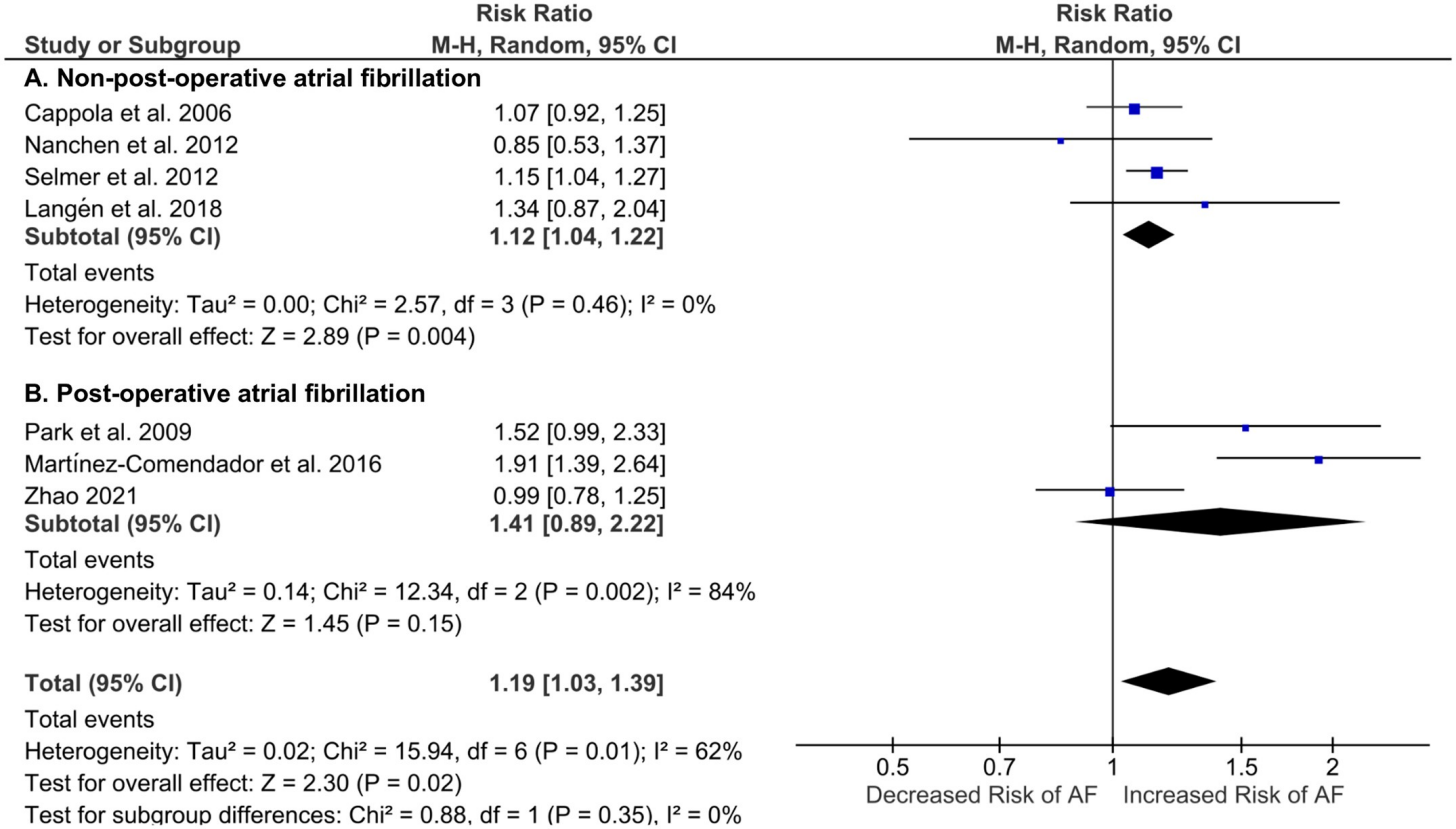
Forest plot of risk ratio for incident atrial fibrillation in patients with subclinical hypothyroidism. Risk ratios for non-post-operative (A) and post-operative (B) atrial fibrillation (AF) in this meta-analysis and overall effect sizes are shown. The size of blue squares corresponds with the weighting of the study and bars represent 95% confidence intervals. Risk ratio expresses risk of AF occurring in patients with subclinical hypothyroidism relative to euthyroid patients. The black diamonds denote effect sizes for non-postoperative and post-operative AF subgroups.

Seven studies assessed the effect of subclinical hypothyroidism on the incidence of AF [29, 30, 33–37]; 727 cases of incident AF were reported among 13,628 patients with subclinical hypothyroidism. Meta-analysis (7 studies) revealed a pooled RR 1.19 (95% CI, 1.03–1.39; *p* = 0.02) for incident AF (Fig 3), although substantial heterogeneity was evident (*I*^2^ = 62%). Subgroup analysis was performed for non-post-operative and post-operative AF. The RR for developing non-post-operative AF in patients with subclinical hypothyroidism was 1.12 and heterogeneity was low (95% CI, 1.04–1.22; n = 4 studies; *p* = 0.004; *I*^2^ = 0%) (Fig 3A). RR for post-operative AF was higher at 1.41 (95% CI, 0.89–2.22; n = 3 studies; *p* = 0.15; *I*^2^ = 84%) (Fig 3B), although this was not statistically significant and with high heterogeneity. The study by Martinez-Comendador *et al*. involved patients undergoing aortic valve replacement surgery and the reported RR was 1.91 (95% CI, 1.39–2.64; *p*<0.0001). Park *et al*. and Zhao *et al*. involved patients undergoing CABG procedure and their combined RR was 1.18 (95% CI, 0.78–1.80; *p* = 0.43; *I*^2^ = 67%).

### Sensitivity analysis

Sensitivity analysis was performed using the leave-one-out approach (Table 2). There was substantial heterogeneity in studies examining the relationship between subclinical hyperthyroidism and incident AF (*I*^2^ = 67%). The removal of Nanchen *et al*. [33] reduced the heterogeneity (*I*^2^ = 19%) but the results were consistent, albeit with a slightly higher risk of incident AF with subclinical hyperthyroidism, RR was 2.28 (95% CI, 1.96–2.65; n = 4 studies; *p*<0.00001). Heterogeneity was not observed in the assessment of the relationship between subclinical hypothyroidism and non-post-operative AF (*I*^2^ = 0%). In the sensitivity analysis for subclinical hypothyroidism and post-operative AF, removing Zhao *et al*. [37] significantly reduced the heterogeneity (*I*^2^ = 0%) and revealed a significant relationship between subclinical hypothyroidism and post-operative AF, RR was 1.76 (95% CI, 1.36–2.28; n = 2 studies; *p*<0.0001).

**Table 2:** Results of sensitivity analyses by leave-one-out method.

**Subclinical hyperthyroidism and incident AF**			
**Studies removed**	**RR [95% CI]**	***I*^2^ (*p* value)**	**Test for overall effect**
None	1.99 [1.43, 2.77]	67% (*p* = 0.02)	Z = 4.05 (*p*<0.0001)
Tenerz *et al*. 1990 [28]	1.95 [1.38, 2.74]	74% (*p* = 0.009)	Z = 3.83 (*p* = 0.0001)
Cappola *et al*. 2006 [29]	1.92 [1.11, 3.32]	68% (*p* = 0.02)	Z = 2.33 (*p* = 0.02)
Nanchen *et al*. 2012 [33]	2.28 [1.96, 2.65]	19% (*p* = 0.30)	Z = 10.57 (*p*<0.00001)
Selmer *et al*. 2012 [34]	1.66 [0.90, 3.08]	68% (*p* = 0.02)	Z = 1.61 (*p* = 0.11)
Langén *et al*. 2018 [36]	1.81 [1.16, 2.81]	75% (*p* = 0.007)	Z = 2.61 (*p* = 0.009)
**Subclinical hypothyroidism and non-post-operative AF**			
**Studies removed**	**RR [95% CI]**	***I***^**2**^ **(*p* value)**	**Test for overall effect**
None	1.12 [1.04, 1.22]	0% (*p* = 0.46)	Z = 2.89 (*p* = 0.004)
Cappola *et al*. 2006 [29]	1.14 [1.04, 1.26]	1% (*p* = 0.36)	Z = 2.64 (*p* = 0.008)
Nanchen *et al*. 2012 [33]	1.13 [1.05, 1.23]	0% (*p* = 0.55)	Z = 3.04 (*p* = 0.002)
Selmer *et al*. 2012 [34]	1.07 [0.94, 1.23]	0% (*p* = 0.38)	Z = 1.04 (*p* = 0.30)
Langén *et al*. 2018 [36]	1.12 [1.03, 1.21]	0% (*p* = 0.39)	Z = 2.68 (*p* = 0.007)
**Subclinical hypothyroidism and post-operative AF**			
**Studies removed**	**RR [95% CI]**	***I***^**2**^ **(*p* value)**	**Test for overall effect**
None	1.41 [0.89, 2.22]	84% (*p* = 0.002)	Z = 1.45 (*p* = 0.15)
Park *et al*. 2009 [30]	1.37 [0.69, 2.69]	91% (*p* = 0.0007)	Z = 0.90 (*p* = 0.37)
Martínez-Comendador *et al*. 2016 [35]	1.18 [0.78, 1.80]	67% (*p* = 0.08)	Z = 0.78 (*p* = 0.43)
Zhao *et al*. 2021 [37]	1.76 [1.36, 2.28]	0% (*p* = 0.39)	Z = 4.32 (*p*<0.0001)

AF: atrial fibrillation; RR: risk ratio; CI: confidence interval.

### Risk of bias and quality of evidence

Using the RoBANS tool and a classification criterion (S2 Table), six studies were assessed as high risk across three RoBANS domains [28–30, 33–35]. One study each was deemed high risk under ‘selection of participants’ and ‘confounding variables’ domains due to small sample size (<100 participants) [28] and inclusion of patients taking thyroid hormone supplement at baseline, respectively [33]. Two studies were classed as unclear risk under ‘confounding variables’ domain as there was no mention of whether patients were taking thyroid medication at baseline [28, 35]. Five studies were rated high risk under the ‘exposure measurement’ domain as they did not measure free T3 levels [29, 30, 33–35]; and one study was assessed unclear risk as free T4 and TSH were measured, but there was no indication of whether T3 was measured [36]. The overall risk of bias assessment identified seven studies [29–32, 34, 36] with low overall risk and three studies [28, 33, 35] with medium overall risk of bias (Table 3). Funnel plots for studies investigating the relationship between subclinical thyroid dysfunction and incident atrial fibrillation are shown (S1 Fig).

**Table 3:** Risk of bias assessment for included studies using the RoBANS tool.

Study	Selection of participants	Confounding variables	Exposure measurement	Blinding of outcome assessment	Incomplete outcome data	Selective outcome reporting	Overall risk of bias
Tenerz *et al*. 1990 [28]	High risk	Unclear risk	Low risk	Low risk	Low risk	Low risk	Medium risk
Cappola *et al*. 2006 [29]	Low risk	Low risk	High risk	Low risk	Low risk	Low risk	Low risk
Park *et al*. 2009 [30]	Low risk	Low risk	High risk	Low risk	Low risk	Low risk	Low risk
Rosario 2010 [31]	Low risk	Low risk	Low risk	Low risk	Low risk	Low risk	Low risk
Poola *et al*. 2011 [32]	Low risk	Low risk	Low risk	Low risk	Low risk	Low risk	Low risk
Nanchen *et al*. 2012 [33]	Low risk	High risk	High risk	Low risk	Low risk	Low risk	Medium risk
Selmer *et al*. 2012 [34]	Low risk	Low risk	High risk	Low risk	Low risk	Low risk	Low risk
Martínez-Comendador *et al*. 2016 [35]	Low risk	Unclear risk	High risk	Low risk	Low risk	Low risk	Medium risk
Langén *et al*. 2018 [36]	Low risk	Low risk	Unclear risk	Low risk	Low risk	Low risk	Low risk
Zhao *et al*. 2021 [37]	Low risk	Low risk	Low risk	Low risk	Low risk	Low risk	Low risk

The quality of the evidence regarding the effects of subclinical hyperthyroidism and subclinical hypothyroidism on AF was low as all included studies were observational studies with a prospective or retrospective cohort design. The GRADE summary of findings is presented in Table 4.

**Table 4:** Quality of evidence assessment by GRADE.

**The effect of subclinical hyperthyroidism on incidence of atrial fibrillation (AF)**					
**Intervention**: Subclinical hyperthyroidism					
**Comparison**: Euthyroid					
**Outcomes**	Anticipated absolute effects* (95% CI)		**Relative effect (95% CI)**	**№ of participants (studies)**	**Certainty of the evidence (GRADE)**
	**Risk with euthyroid**	**Risk with subclinical hyperthyroidism**			
AF	31 per 1,000	**61 per 1,000**	**RR 1.99**	581329	⨁⨁◯◯
		(44 to 86)	(1.43 to 2.77)	(5 observational studies)	LOW
**The effect of subclinical hypothyroidism on the incidence of atrial fibrillation**					
**Intervention**: Subclinical hypothyroidism					
**Comparison**: Euthyroid					
**Outcomes**	Anticipated absolute effects* (95% CI)		**Relative effect (95% CI)**	**№ of participants (studies)**	**Certainty of the evidence (GRADE)**
	**Risk with euthyroid**	**Risk with subclinical hypothyroidism**			
AF	31 per 1,000	**37 per 1,000**	**RR 1.19**	589590	⨁⨁◯◯
		(32 to 44)	(1.03 to 1.39)	(7 observational studies)	LOW
Non-post-operative AF	31 per 1,000	**35 per 1,000**	**RR 1.12**	587786	⨁⨁◯◯
		(32 to 38)	(1.04 to 1.22)	(4 observational studies)	LOW
Post-operative AF	256 per 1,000	**361 per 1,000**	**RR 1.41**	1804	⨁⨁◯◯
		(228 to 569)	(0.89 to 2.22)	(3 observational studies)	LOW
*The risk in the intervention group (and its 95% confidence interval) is based on the assumed risk in the comparison group and the **relative effect** of the intervention (and its 95% CI).					
**CI**: Confidence interval; **RR**: Risk ratio					
**GRADE Working Group grades of evidence**					
**High certainty**: We are very confident that the true effect lies close to that of the estimate of the effect					
**Moderate certainty**: We are moderately confident in the effect estimate: The true effect is likely to be close to the estimate of the effect, but there is a possibility that it is substantially different					
**Low certainty**: Our confidence in the effect estimate is limited: The true effect may be substantially different from the estimate of the effect					
**Very low certainty**: We have very little confidence in the effect estimate: The true effect is likely to be substantially different from the estimate of effect					

## Discussion

This systematic review assessing the incidence of AF in patients with subclinical thyroid dysfunction identified 10 studies [28–37], all were cohort studies. Meta-analyses revealed that both subclinical hyperthyroidism and subclinical hypothyroidism increased the risk of incident AF by 99% and 19%, respectively. Subclinical hypothyroidism was not found to significantly increase the risk of incident post-operative AF; however, there was considerable heterogeneity and variation in surgical procedures. The quality of current evidence was low for both subclinical hyper- and hypothyroidism, despite low risk of bias in studies.

Patients with subclinical hyperthyroidism had an increased risk of incident AF compared to patients who were euthyroid. Heterogeneity was significant and sensitivity analyses identified one study [33] as the source of heterogeneity; removal of this study also demonstrated an increased risk of incident AF associated with subclinical hyperthyroidism. This study included patients taking thyroid hormone supplements at baseline, which may lead to a reduced risk of incident AF [33]. All other studies in this meta-analysis had either not reported or had excluded patients taking thyroid medication at baseline.

In a systematic review of prospective cohort studies by Sun *et al*., the relationship between subclinical thyroid dysfunction and risk of various cardiovascular outcomes were assessed [39]. Subclinical hyperthyroidism was associated with increased risk of incident AF (RR, 1.42; 95% CI, 0.69–2.92; *p* = 0.025; *I*^2^ = 72.9%). However, their meta-analysis was limited by small sample size (3 studies with one study being unpublished data), wider 95% CI and significant heterogeneity [39]. Our analysis is updated and includes a larger number of studies (n = 5 including the two published studies analysed by Sun *et al*.), with tighter 95% CI (1.43–2.77). Additionally, our finding that subclinical hyperthyroidism increased the risk of incident AF is consistent with a 2012 systematic review by Collet *et al*. that reported a hazard ratio of 1.68 (95% CI; 1.16–2.43) [40].

The association between subclinical hyperthyroidism and AF was recently reviewed [41]. This narrative review included cross-sectional studies and case-controls studies in which thyroid hormone levels were measured after AF diagnosis [41]. Our PROSPERO protocol stipulated within the inclusion criteria that thyroid status was determined at baseline before the AF event to determine whether subclinical thyroid status leads to AF. All included studies were cohort studies, and a causal relationship could not be determined. Overall, our analysis showed that in longitudinal observational studies patients with subclinical hyperthyroidism are more likely to have incident AF than euthyroid patients.

The present systematic review demonstrates that patients with subclinical hypothyroidism also have increased risk of AF, although substantial heterogeneity was evident. A subgroup analysis for non-post-operative and post-operative AF eliminated heterogeneity (*I*^2^ = 0) in the non-post-operative AF group and demonstrated a slightly increased risk of incident AF in this group. In contrast, two previous systematic reviews assessing the relationship between subclinical hypothyroidism and non-post-operative incident AF did not report significant associations [39, 42]. One [42] was an individual patient data analysis of prospective cohorts and excluded patients taking thyroid hormone at baseline, whilst the other [39] was limited by inclusion of only two studies. The present analyses included prospective [28–31, 33, 35, 36] and retrospective [32, 34, 37] cohort data and patients taking thyroid hormone at baseline, which may explain the differences in RRs reported.

To date this is the first systematic review and meta-analysis to have assessed the relationship between subclinical hypothyroidism and incident post-operative AF. AF presenting post-operatively is mostly transient [18, 30, 35], but is associated with prolonged hospital stays, an 8-fold increased risk of developing AF, and a doubling of long-term cardiovascular mortality [43, 44]. The present analysis showed no significant difference in the overall risk of post-operative AF associated with subclinical hypothyroidism, however there was significant heterogeneity. Among the different surgical procedures, risk of incident AF was significantly elevated post-aortic valve replacement [35], but two studies of CABG patients [30, 37] reported conflicting results. The prospective cohort study [30] (n = 260) reported a significant risk of post0operative incident AF [30], while the other retrospective cohort study [37], found no difference. The strength of prospective cohort study is the accuracy of data collection (for e.g., exposures, confounders and endpoints), with fewer potential sources of bias and confounders, prospective cohort design is typically ranked higher than retrospective cohort design in the hierarchy of evidence [45]. The conflicting results about subclinical hypothyroidism and post-operative AF might also reflect differences in the patient demographics and geographical location of the population studied. Additionally, participants in the prospective study had multiple thyroid hormone tests before and after CABG surgery [30], whereas only one pre-surgery blood test result was used in the retrospective study [37], which may have resulted in a misclassification bias. Sensitivity analyses identified the retrospective study as a source of heterogeneity; removal of this study revealed a significant association between subclinical hypothyroidism and increased risk of post-operative AF. Short term changes in levels of circulating thyroid hormones occur during cardiac surgery because of the surgical insult and may alter atrial electrophysiology leading to post-operative AF [46]. Some evidence suggests a role for postoperative T3/T4 treatment to prevent post-operative AF in cardiac surgery patients [46–48].

In the current systematic review, all included studies were observational and confounding factors were not consistently adjusted for. Therefore, it is possible that confounders including sex, age, ethnicity, pre-existing co-morbidities, and other unmeasured confounders could have affected AF incidence. Two of the 10 included studies had a mean/median follow-up time >10 years [29, 36]; longer follow-up times can result in higher AF incidence [49]. For subclinical hyperthyroidism, the results of these studies were not dissimilar to those of other studies with shorter follow time periods (2 to 5.5 years) [28, 33, 34]. For subclinical hypothyroidism, one of the two studies with >10-year follow-up [36] demonstrated the highest risk for incident AF (non-post-operative). However, the other study [29] reported AF incidence that was similar to those with shorter follow-up times (3 to 5.5 years) [33, 34]. The difference in mean participant age between the two studies (50.9 *vs*. 72.7 years) is a likely confounding factor [49, 50]. Currently, there is insufficient enough evidence to determine conclusively if there is increased risk of incident AF in patients with subclinical hypothyroidism over a longer follow-up period.

A major strength of our systematic review is the comprehensive literature search from database inception, without language restrictions, for RCTs, case-control, and cohort studies. Previous systematic reviews have only included prospective cohort studies [39, 42]. Our review was registered with PROSPERO which involved prospective submission and publication of information relating to the design and conduct of a systematic review. We had a large participant size which increased the precision of the results. Robust methodology was employed with two reviewers independently screening for eligible studies and dual data extraction. Further, this review utilised the GRADE approach to assess the quality of the evidence, which demonstrated low quality evidence. Furthermore, we assessed the risk of incident AF in both subclinical hyper- and hypothyroidism using a uniform approach.

One limitation of this review was the varying definitions used for subclinical thyroid status in the included studies would likely have resulted in some participants being assigned to euthyroid groups in certain studies but to subclinical thyroid dysfunction groups in other studies. An individual patient data analysis would have avoided this problem but was not conducted due to non-availability of data. AF can be paroxysmal, and it is plausible that in patients with subclinical thyroid dysfunction, undiagnosed AF may have pre-existed, and the recorded incident events may not have been new-onset AF.

## Conclusions

This systematic review and meta-analysis suggests that both subclinical hyperthyroidism and subclinical hypothyroidism are associated with increased risk of incident AF; however, the quality of the available evidence is low. Future studies should have consistency in the definition of subclinical hyper- and hypothyroidism, RCT design, large sample size and long follow-up to allow assessment of the effect of treatment on the incidence of AF and other cardiovascular outcomes in patients with subclinical thyroid dysfunction.

## Supporting information

S1 Table Search strategy for Scopus and MEDLINE databases. (PDF)

S2 Table Risk of bias classification criteria for RoBANS domains. (PDF)

S1 Fig Funnel plot for studies on the relationship between subclinical thyroid dysfunction and incident atrial fibrillation. (A) Subclinical hyperthyroidism and incident atrial fibrillation (AF), (B) subclinical hypothyroidism and incident AF and post-operative AF. The x-axis represents the risk ratio (RR) and y-axis points to the standard error of the RR on a logarithmic scale. Data for non-post-operative AF (black circle) and post-operative AF (red diamond) are shown.
(PDF)

S1 Checklist PRISMA 2009 checklist. (PDF)
