# Supplementary material for: Subclinical thyroid dysfunction and the risk of incident atrial fibrillation: A systematic review and meta-analysis
Source: PLoS One. 2024 Jan 2;19(1):e0296413. doi: 10.1371/journal.pone.0296413 (PMC10760776; doi:10.1371/journal.pone.0296413)
Supplement: S1 Table — (PDF) [file pone.0296413.s001.pdf]

Supplementary Table 1: Search strategy for Scopus and MEDLINE databases.

**Scopus**	**MEDLINE**	
TITLE-ABS-KEY ("subclinical hyperthyroidism" OR "subclinical hypothyroidism" OR "subclinical thyroid" OR "thyroid dysfunction" OR "thyroid")	1	subclinical hyperthyroidism.mp
	2	subclinical hypothyroidism.mp
	3	subclinical thyroid.mp
	4	thyroid dysfunction.mp
	5	thyroid.mp
AND	6	1 OR 2 OR 3 OR 4 OR 5
	7	atrial fibrillation.mp
TITLE-ABS-KEY ("atrial fibrillation" OR "arrhythmia")	8	arrhythmia.mp
	9	7 OR 8
	10	6 and 9
