# Supplementary material for: Subclinical thyroid dysfunction and the risk of incident atrial fibrillation: A systematic review and meta-analysis
Source: PLoS One. 2024 Jan 2;19(1):e0296413. doi: 10.1371/journal.pone.0296413 (PMC10760776; doi:10.1371/journal.pone.0296413)
Supplement: S2 Table — (PDF) [file pone.0296413.s002.pdf]

Supplementary Table 2: Risk of bias classification criteria for RoBANS domains.

RoBANS domain	Risk	Criteria
**Selection of participants**	Low	>100 participants in the study
	High	<100 participants in the study
**Confounding variable**	Low	Patients taking thyroid medication were excluded from their analysis
	High	Patients were taking thyroid medication at baseline, and this was not accounted for
	Unclear	No mention of whether patients were taking thyroid medication at baseline
**Exposure measurement**	Low	TSH, free T4, and free T3 were measured at baseline
	High	Free T3 was not measured
	Unclear	Free T4 and TSH measured; no indication whether T3 was measured
**Blinding of outcome assessment**	Low	AF is an objective diagnosis and blinding does not play a major role
	High	NA
**Incomplete outcome data**	Low	AF outcome data is reported for patients with subclinical hyperthyroidism, subclinical hypothyroidism and euthyroidism, where appropriate
	High	AF outcome data is not reported
**Selective outcome reporting**	Low	RoBANS tool is used to assess the bias of an outcome (AF), rather than the entire study hence it is low risk
	High	NA

TSH: thyroid stimulating hormone; T4: tetraiodothyronine; T3: triiodothyronine; AF: atrial fibrillation; NA: not applicable.
