# Supplementary figures and images for: Subclinical thyroid dysfunction and the risk of incident atrial fibrillation: A systematic review and meta-analysis
Source: PLoS One. 2024 Jan 2;19(1):e0296413. doi: 10.1371/journal.pone.0296413 (PMC10760776; doi:10.1371/journal.pone.0296413)

### A. Subclinical hyperthyroidism and incident AF.

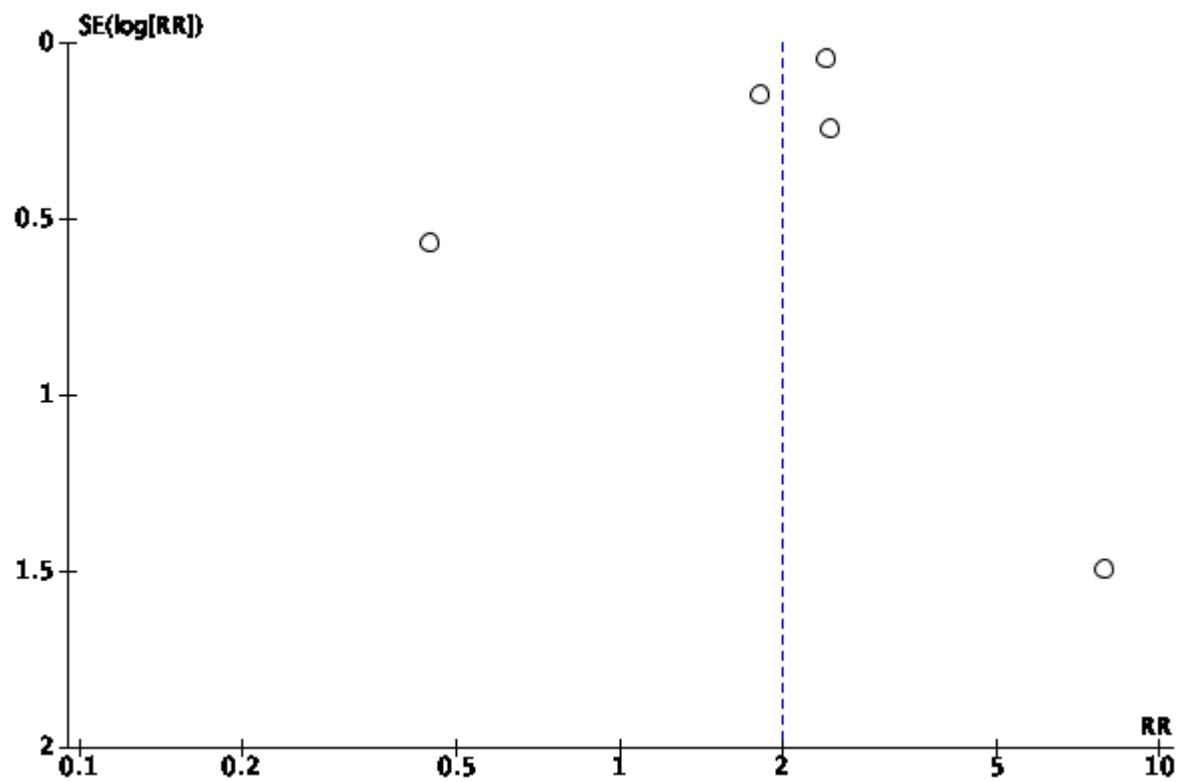

### B. Subclinical hypothyroidism and incident AF.

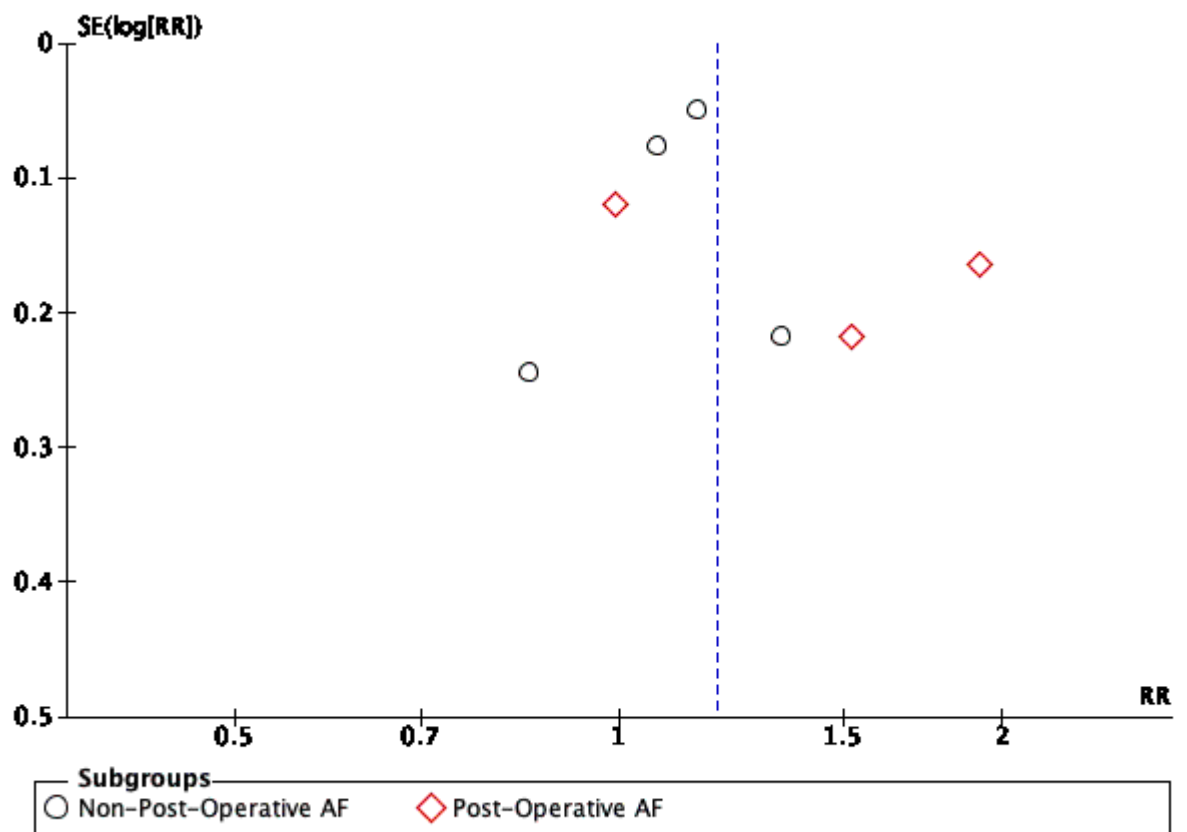

Supplement: S1 Fig — (A) Subclinical hyperthyroidism and incident atrial fibrillation (AF), (B) subclinical hypothyroidism and incident AF and post-operative AF. The x-axis represents the risk ratio (RR) and y-axis points to the standard error of the RR on a logarithmic scale. Data for non-post-operative AF (black circle) and post-operative AF (red diamond) are shown. (PDF) [file pone.0296413.s003.pdf]
